# Chromosome-level genome assembly of Xuefeng Black-bone chicken and comparative genomics analysis

**DOI:** 10.1186/s12864-026-12952-z

**Published:** 2026-05-20

**Authors:** Songchang Guo, Jingzhe Huang, Qiongtao Zi, Peng Li, Lingyun He, Xi He, Haihan Zhang, Donghua Li, Meichun Li, Xiangyong Qu, Changqing He

**Affiliations:** 1 College of Animal Science and Technology, Hunan Agricultural University, Changsha, Hunan 410128 China; 2 Yuelushan Laboratory, Changsha, Hunan 410128 China; 3 Huaihua Animal Husbandry and Fishery Affairs Center, Huaihua, Hunan 418000 China; 4 College of Animal Science and Technology, Henan Agricultural University, Zhengzhou, Henan 450002 China; 5 Hunan Yunfeifeng Agricultural Co. Ltd., Huaihua, Hunan 418200 China

**Keywords:** Xuefeng Black-bone chicken, Genome assembly, Genome annotation, Comparative genomics analysis

## Abstract

**Background:**

Xuefeng Black-bone chicken is a precious poultry variety for its characteristic traits, high-economic value and medicinal diet value. However, the genomic information for the Xuefeng Black-bone chicken was still lacking, which have hindered the identification of genetic variants underlying its distinctive traits and impeded the implementation of genomic selection strategies in breeding programs. Therefore, we applied PacBio SMRT (Single Molecule Real-Time) sequencing, Illumina sequencing, Hi-C and RNA-seq to establish a high-contiguity reference genome for Xuefeng Black-bone chicken, achieving chromosome-level.

**Results:**

The de novo assembly yielded a 1.13 Gb genome, exhibiting contiguity metrics of 21.76 Mb (contig N50) and 83.79 Mb (scaffold N50), and comprised 40 pseudochromosomes. Merqury demonstrated a QV (Consensus quality value) score of 43.1876 and 96.7% BUSCO gene representation completeness. The genome of the Xuefeng Black-bone chicken contained about 18.48% repetitive sequences. The genomic annotation identified 20,146 protein-coding genes, of which 15,804 (78.45%) possessed functional characterization, and 1471 ncRNAs (non-coding RNAs) were also predicted. Comparative genomics analysis revealed 5,170,902 SNPs (single nucleotide polymorphisms) and 1127 SVs (structural variations) between Xuefeng Black-bone chicken genome and other chicken varieties genomes. These SVs were linked to 11,391 genes enriched in transcription by RNA polymerase II, heterocycle biosynthetic process, DNA binding, peroxisome and calcium signaling pathway, which mainly involved in the environmental adaption, stress resistance and immune system.

**Conclusions:**

Our assembly of the Xuefeng Black-bone chicken genome was high quality and the genome will provide more resources and insights into the unique characteristics study of the chicken, which will enhance our understanding and guide the breeding work better.

**Graphical Abstract:**

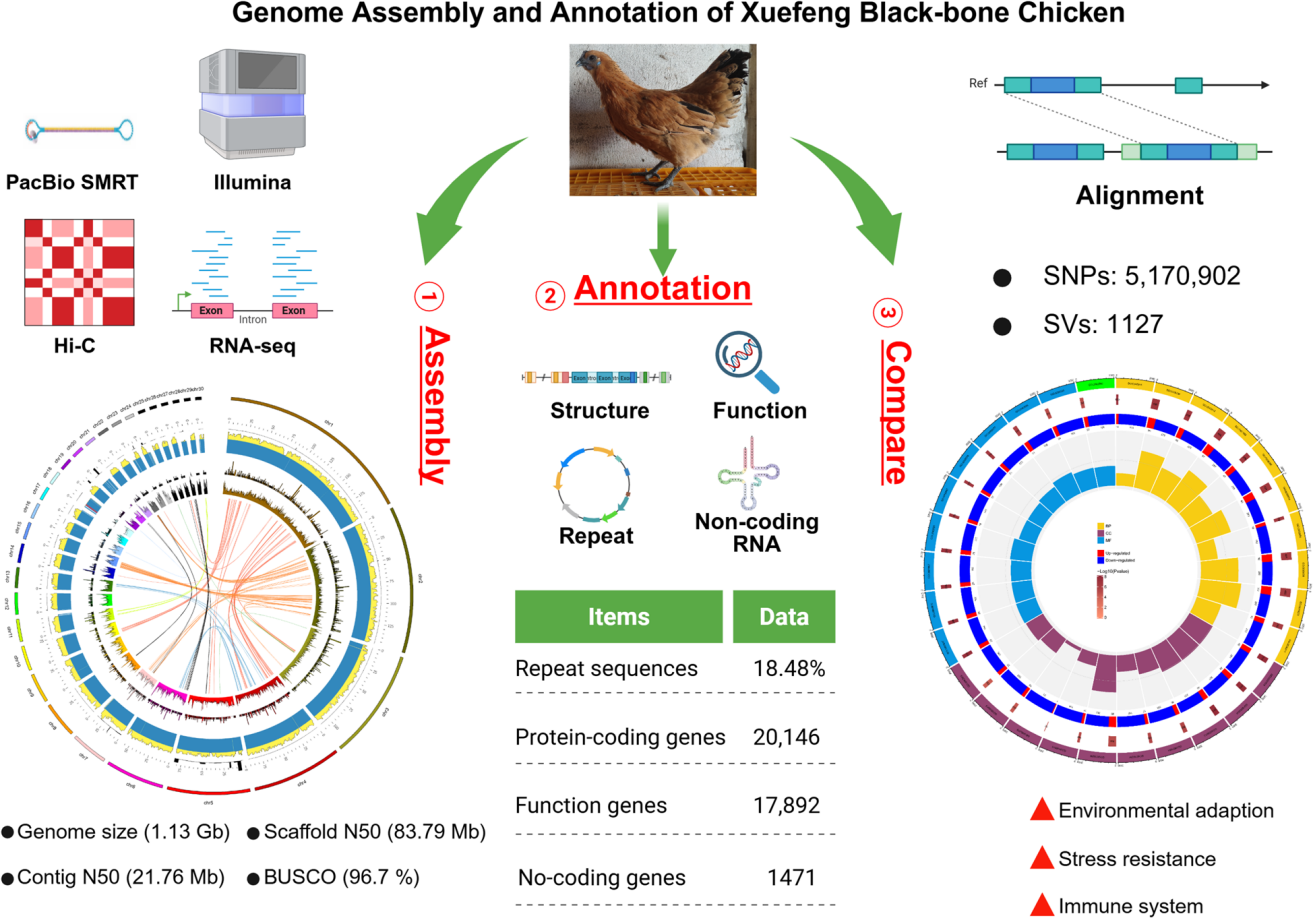

**Supplementary Information:**

The online version contains supplementary material available at 10.1186/s12864-026-12952-z.

## Background

The genome encompasses all genetic information of an organism, including both the nuclear genome and organelle genome. The reference genome of a species provides essential genotype and regulatory element data, which are crucial for elucidating the mechanisms underlying the formation of its characteristic phenotypes or diseases of interest. Consequently, the genome assembly of a species has significant implications for advancing its genetic and breeding research. Early genome assembly was conducted using clone maps, short-length reads or other mapping data, which resulted in low genome quality, high costs, and extended processing times. Advancements in long-read sequencing technology now make it possible to achieve nearly complete assembly of each chromosome [1, 2] and even telomere-to-telomere assembly [3].

In recent years, rapid advancements in sequencing technologies and bioinformatics have also significantly contributed to the development of genomic resources for domestic chickens. Since the first draft assembly of the chicken genome was released in 2004, substantial advancements in chicken genomics have been achieved. These developments have provided valuable data that benefit both the poultry industry and biomedical research [4]. The GRCg7b, the most recent reference genome for chickens, originated from a hybrid male whose mother was a broiler and father a layer, and the genome was constructed utilizing long sequencing reads in combination with various scaffolding data [5, 6]. Compared with the initial draft genome assembly of a red jungle fowl individual, the completeness, continuity and accuracy of the GRCg7b have been greatly improved [5]. Meanwhile, there were several assemblies for indigenous chickens released in GenBank, including Wenchang chicken (ASM4043664v1 and ASM4043665v1), Piao chicken (ASM3091426v2), Daweishan chicken (ASM3084955v2), Wuding chicken (ASM3091427v2) and Silkie chicken (ASM2465302v1). These assemblies constructed a solid basis for recognizing different aspects of chicken biology, domestication, and evolution, as well as guiding poultry breeding practices.

The Xuefeng Black-bone chicken, originating from the Xuefeng Mountains region of Huaihua in Hunan Province, China. It is characterized by its 'five black' traits: black skin, meat, bones, beak, and shank. The chicken has a medium body size and robust physique, enabling it to withstand rough feeding and challenging environments. Its meat is tender and flavorful, offering high nutritional value and beneficial medicinal properties, thus preferred by the people. In the early stage, a series of genomic studies were carried out to address problems during the production of Xuefeng Black-bone chickens, including transcriptomics [7, 8], whole genome DNA methylation [9], single-cell nuclear transcriptomics [10]. The screening of numerous related candidate genes offers molecular markers that can be used in the breeding of the chicken. However, the genome assembly and annotation for the Xuefeng Black-bone chicken were still lacking, thus previous bioinformatics analyses of a large amount of sequencing data were based on the Red Jungle fowl genome (GRCg6a) as a reference genome. The research showed that the reference genome choice can influence the bioinformatics analysis [11], and then may have certain limitations in the study of characteristic traits in Xuefeng Black-bone chickens.

Therefore, the current study aimed to combine Illumina sequencing, SMRT (Single Molecule Real-Time) sequencing, Hi-C sequencing, and RNA sequencing to assemble and annotate a genome for the Xuefeng Black-bone chicken. Subsequently, we employed an alignment-based strategy to conduct comparative genomics analysis between the new genome and the genomes of other chicken varieties. The genome assembly generated in this study is expected to supply a reference genome for genomics research and accelerate progress in breeding.

## Methods

### Ethics approval, sample collection and DNA and RNA isolation

All experiments were approved by the Animal Care and Use Committee of College of Animal Science and Technology, Hunan Agricultural University and followed the committee guidelines (Approval Number: HUNAU2023081).

A healthy, adult female and a healthy, adult male Xuefeng Black-bone chicken were collected by hand from Hunan Yunfeifeng Agriculture Co., Ltd., Hongjiang District, Huaihua City, Hunan Province, China (N27.09°, E109.98°) in December 2023. The individuals were chosen from the core breeding flock based on their strict conformity to the breed's standard phenotypic traits and their documented pedigree to ensure breed purity and minimal relatedness. Fresh tissues, including the heart, liver, lung, spleen, brain, kidney and muscle from female chicken, and testis from male chicken, were collected in the liquid nitrogen. Subsequently kept these tissues in the laboratory at −80℃ ultra-low temperature refrigerator until they underwent sequencing. The tissues DNA and RNA were extracted and sequenced at the Novogene Bioinformatics Technology Co., Ltd (Tianjin, China). For genomic DNA extraction, the TIANamp Genomic DNA Kit was employed, and total RNA was isolated using the TIANScript Total RNA Extraction Kit (TIANGEN Biotech, Beijing, China). Nucleic acid quality and quantity were rigorously assessed through Qubit Fluorometer (Thermo Fisher Scientific) and 1% agarose gel electrophoresis.

### Sequencing

Xuefeng Black-bone chicken genome data was acquired through Illumina sequencing, SMRT sequencing, and Hi-C sequencing technologies. DNA was extracted from liver tissue of a female Xuefeng Black-bone chicken for both Illumina and SMRT sequencing. For the Illumina sequencing, a short-read genomic library was constructed utilizing the NEBNext® Ultra™ II DNA Library Prep Kit (New England Biolabs, Ipswich, USA), generating 350 bp insert-sized fragments via enzymatic fragmentation and dual-indexed adapter ligation. Sequencing was performed in 150 bp paired-end mode on an Illumina Novaseq sequencing platform. Raw reads containing > 10% unknown bases or over 20% low-quality bases, as well as all adapter sequences, were removed. For SMRT sequencing, a library with fragment sizes ranging from 15 to 18 kb was processed via the SMRTbell Express Template Prep Kit 2.0 (Pacific Biosciences, CA, USA) and subsequently sequenced on the Pacbio Sequel II/IIe sequencing platform. Raw reads were processed through two steps: the raw reads were broken at the adaptor, and all adaptor sequences were removed, resulting in subreads; the subreads were then processed using CCS software (min-passes = 3, min-rq = 0.99) to generate HiFi reads with quality above Q20. For Hi-C sequencing, the Hi-C library was prepared from muscle sample according to the reference [12]. Then, the library was sequenced on the Illumina NovaSeq 6000 and the low-quality reads were processed via Hi-C-Pro with default parameters.

RNA sequencing was performed on eight tissues (heart, liver, lung, spleen, brain, kidney, muscle, testis). The library was construct via the NEBnext Ultra-Directional RNA Library Prep kit (NEB, protocol B) and sequenced on the Illumina NovaSeq 6000 platform. The low-quality raw reads were filtered using Fastp [13].

### Survey analysis

Before assembly, survey analysis was first implemented to evaluate genome features, including genome size, heterozygosity rate and repetitive sequence rate. The 17-mer depth frequency distribution and total k-mer number were calculated using Jellyfish v2.2.7 [14] and SOAPdenovo2 [15].

Genome size. Based on the following two assumptions, k-mer were extracted base-by-base from the filtered sequencing reads. The k-mer frequency distribution was then counted to calculate the k-mer depth distribution curve and the depth product curve. The estimated k-mer depth derived from these curves was subsequently used to estimate the genome size. Assumption 1: All k-mer extracted base-by-base from each read can traverse the entire genome; Assumption 2: The k-mer depth frequency distribution follows a Poisson distribution.

Heterozygosity rate and repetitive sequence rate. Genome heterozygosity and repetitive sequence rate were inferred from the distribution of k-mer curve. The ratio of the heterozygous peak to the main homozygous peak was used to estimate the heterozygosity rate. The proportion of k-mer with a depth exceeding 1.8 times the main peak depth was used to estimate the repetitive sequence content. This k-mer-based approach provides a preliminary characterization of genome complexity prior to assembly.

### Genome assembly

The genome was assembled using the default parameters of Hifiasm [16]. Hifiasm assembly could be divided into three main steps: (1) The first step was to identify and correct haplotype errors. (2) The second step was the construction of the assembly diagram. (3) The third step was the generation of the assembly sequence. If where no additional data was available, Hifiasm selects one side of each bubble at random to generate the primary contigs, which represent the main assembly output.

The Hi-C sequencing data was used to complete the assisted assembly of a chromosome-level genome [17]. In the present study, we used the ALLHiC software to identify chromosomal Hi-C interactions. The ALLHiC software included five steps: pruning, partitioning, rescue, optimization and building.

### Genome quality assessment

To evaluate the completeness of the Xuefeng Black-bone chicken genome, BUSCO (the aves_odb10 database (Creation date: 2024–01–08)) [18] analysis was performed by searching for 8338 single-copy avian genes. Subsequently, all clean Illumina short reads were mapped to the assembled genome using BWA software [19, 20] to assess the consistency of the genome. Finally, the accuracy was assessed using Merqury software [21]. The Merqury was a *k-mer*-based method, a comparison of the *k-mer* spectrum of short sequencing reads with assembly results in consensus quality value.

### Genome annotation

#### Repetitive element annotation

Repeat elements were annotated using the homology and de novo predictions. For homology, RepeatMasker [22] and its built-in scripts (RepeatProteinMask) were applied with default settings to scan the genome for known repetitive sequences, with the genome sequences used as queries against the reference from the Repbase database [23]. For de novo, RepeatModeler with default parameters was used to construct a de novo repetitive element database, then all repeat sequences with lengths > 100 bp and gap ‘N’ less than 5% included in the raw TE (transposable element) library. Subsequently, using the RepeatMasker [22] to annotate repetitive elements with the database. Finally, a combined library, consisting of Repbase and the de novo TE library processed through UCLUST to remove redundancy, was provided to RepeatMasker for DNA-level repeat identification. Tandem repeats were annotation with Tandem Repeat Finder (TRF).

#### Annotation of protein-coding genes

Gene prediction was performed using a combination of methods, including homology, de novo, and transcriptome approaches. For homology-based prediction, homologous proteins sequences data of GRCg6a (*Gallus gallus*, GCF_000002315.6), GRCg7b (*bGalGal1.mat.broiler.GRCg7b*, GCF_016699485.2), goose (*Anser cygnoides*, GCF_000971095.1) and Pekin duck (*Anas platyrhynchos*, GCF_015476345.1) were obtained from NCBI and then were aligned to the genome using TBLASTN (E-value ≤ 10^−5^) [24]. Subsequently, the identified proteins were matched to homologous genome sequences for precise spliced alignments using GeneWise [25]. For de novo prediction, Augustus [26] and SNAP [27] were used in our automated gene prediction pipeline. For transcriptome-based prediction, transcriptome reads assemblies were generated with Trinity [28] for the genome annotation. To enhance genome annotation accuracy, the RNA-Seq reads from different tissues were aligned to genome fasta using Hisat [29] with default parameters to identify exons region and splice positions. The alignment results were then used as input for Stringtie [30] with default parameters for genome-based transcript assembly. The non-redundant reference gene set was generated by merging genes predicted by three methods with EvidenceModeler (EVM) [31] using PASA (Program to Assemble Spliced Alignment) terminal exon support and including masked transposable elements as input into gene prediction.

#### Functional annotations

Gene functions were annotated based on the best match by aligning the protein sequences to the Swiss-Prot using Blastp (with a threshold of E-value ≤ 10^−5^). To annotate motifs and domains, InterProScan [32] was employed through searches across various public databases like ProDom, PRINTS, Pfam, SMRT, PANTHER, and PROSITE. The GO (Gene Ontology) IDs for each gene were assigned according to the corresponding InterPro entry. The prediction of protein functions involved transferring annotations from the nearest BLAST hit (E-value < 10^–5^) in the Swissprot database [33] and DIAMOND/BLAST hit (E-value < 10^–5^) in the NR database [33]. Furthermore, a gene set was mapped to a KEGG (Kyoto Encyclopedia of Genes and Genomes) pathway, and the best match for each gene was identified.

To maximize the reliability of the gene annotation, we implemented a post-filtering pipeline. The specific criteria are as follows: 1) Any gene model whose overlap with TE sequences covered ≥ 20% of its length was removed to eliminate sequences potentially derived from transposable elements; 2) Genes containing premature stop codons or ambiguous nucleotides ('N') within their CDS were discarded; 3) Genes supported solely by de novo evidence with RPKM values less than 1 across all tissues were removed; 4) Single-exon genes lacking both homology and transcriptome support, and with an RPKM value less than 5, were removed. Furthermore, we adopted the conservative framework for identifying reliable novel genes as described by Li et al. [34]. Briefly, gene redundancy from de novo and reference-guided annotations was reduced using CD-HIT (-c 0.9 -aS 0.8 -d 0 -sf 1). The remaining genes were then aligned against the nr database and the GRCg6a genome using BLASTN 2.6.0 +. Genes with no significant hits to either non-Chordata sequences or GRCg6a were defined as completely novel. Genes showing > 95% identity to GRCg6a genes were classified as partially missing in the reference genome. Finally, we removed all predicted genes with a total gene length of less than 1 kb.

#### Non-coding RNAs annotations

The tRNAs were predicted using the program tRNAscan-SE [35]. As rRNAs are highly conserved, we identified the rRNA by mapping chicken rRNA sequences to the Xuefeng Black-bone chicken genome. miRNAs and snRNAs were identified by searching against the Rfam database [36] with default parameters using the infernal software.

### Comparative genomics analysis

#### Synteny analysis

Synteny analysis of genomes was performed via whole-genome alignment using MUMmer4 [37] for Xuefeng Black-bone chicken genome versus GRCg7b, and Xuefeng Black-bone chicken genome versus GRCg6a. Genome alignment was conducted by NUCmer, and then the alignment block filter was applied using a delta-filter with one-to-one alignment mode. The homologous genes were analyzed by the MCScanX package [38] with default settings, except for gap_penalty-3. Syntenic blocks were characterized as those containing a minimum of five syntenic genes.

#### SNPs analysis

To compare the genome difference between the Xuefeng Black-bone chicken and GRCg6a, we performed the SNPs (Single nucleotide polymorphisms) analysis using the Nucmer and Show-snp programs of MUMmer [37].

#### Structural variations analysis

We conducted the SVs (Structural variations) analysis between the Xuefeng Black-bone chicken genome and other chicken genome according to the reference [39]. The assembly data of other nine chickens (GCA_024206055.1 (Huxu), GCA_024652995.1 (White Leghorn), GCA_024653025.1 (Silkie), GCA_024652985.1 (Rhode Island Red), GCA_024653045.1 (Houdan), GCA_024653035.1 (Cornish), GCA_040436645.1 (Wenchang), GCA_030849555.2 (Daweishan) and GRCg7b) was downloaded from NCBI. All assemblies were aligned to Xuefeng Black-bone chicken genome using Winnowmap2 [40]. Svim-asm [41] was used to detect variations with haploid mode and SURVIVOR [42] was used to detect hitherto unknown assembled sequences. The absence was determined by deletion variation with a length over 50 bp in the resulting VCF file.

#### GO and KEGG enrichment analyses

GO enrichment analysis was conducted using the GOseq R package. Statistical enrichment of genes in KEGG pathways was assessed using the KOBAS 3.0 software (http://kobas.cbi.pku.edu.cn/kobas3/download/).

Detailed information on the software and parameters used in this study is provided in the Supplementary Supplementary Material 10.

## Results

### Survey analysis

A total of 84.68 Gb whole genome short reads was produced, with 66.95 Gb high-quality data retained after Fastp filtering (Supplementary Material 3). Results showed that the total *k-mer* number was 59,743,616,398 and the peak *k-mer* frequency depth of *17-mer* was 55x (Supplementary Material 1). Finally, survey analysis revealed a 1.07 Gb assembly size genome characterized by 0.65% heterozygosity and 35.13% repetitive elements (Supplementary Material 4).

### Genome assembly and evaluation

**Fig. 1:**
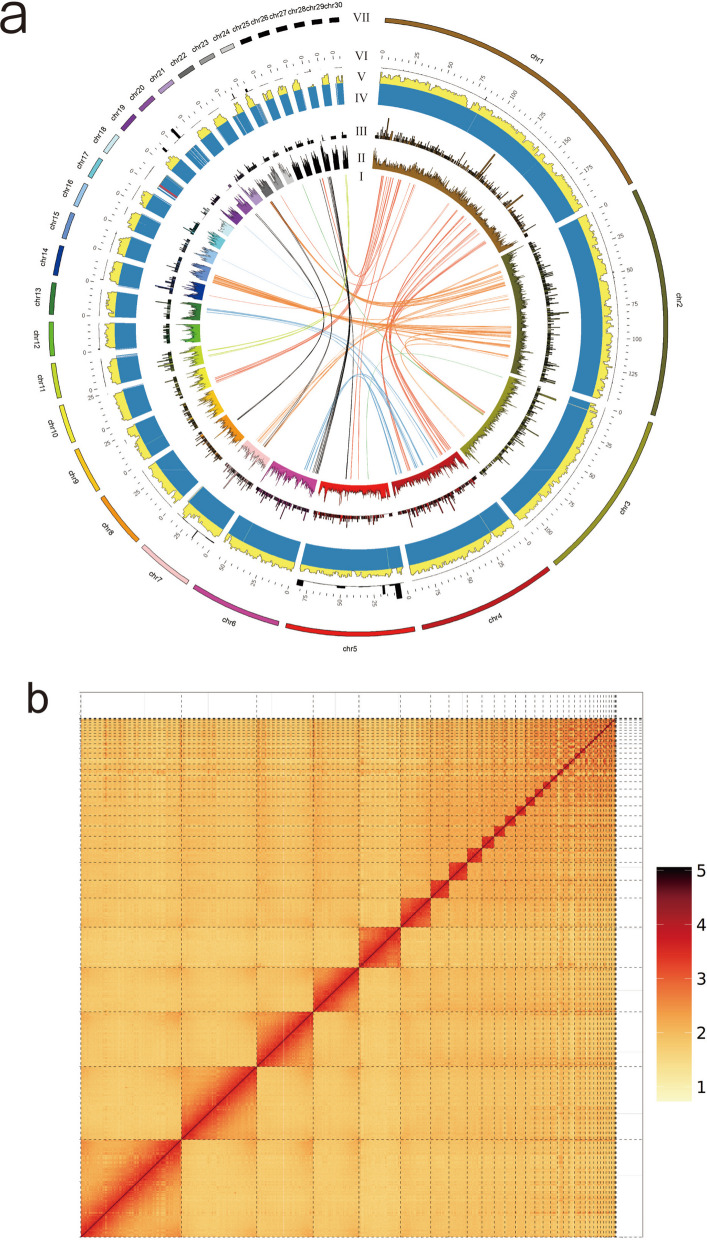
Features of Xuefeng Black-bone chicken genome. Genome Assembly and Evaluation. **a** Features of Xuefeng Black-bone chicken genome. From inner to outer circles: (I) Syntenic regions containing more than four paralogous genes, (II) Guanine-cytosine (GC) content, (III) Gene distribution, (IV) Transposable element (TE) density, (V) SV content, (VI) SNP content, (VII) Lines represent Xuefeng Black-bone chicken pseudochromosomes. **b** Hi-C-based heatmaps of DNA interaction of each chromosome (500 K). X-axis represents pseudochromosomes, Y-axis represents pseudochromosomes. Each square from the bottom left corner to the top right corner represents chr1, chr2, chr3, chr4, chrZ, chr5, chr6, chr7, chr8, chr9, chr12, chr10, chr11, chr13, chr14, chr20, chr15, chrW, chr18, chr29, chr19, chr17, chr28, chr27, chr24, chr21, chr23, chr31, chr26, chr22, chr16, chr25, chr30, chr32, chr33, chr34, chr35, chr36, chr37, chr38

Following CCS-based quality refinement, 49.66 Gb (45.73x) HiFi reads generated from PacBio Sequel II sequencing (read length N50 = 17.221 kb) were processed through Hifiasm, yielding a primary assembly (1.13 Gb) with contig N50 = 21.76 Mb and scaffold N50 = 83.79 Mb (Supplementary Material 3 and Material 9). Hi-C scaffolding using 108.49 Gb filtered data from 150.41 Gb raw reads (138.50x) enabled ALLHiC-directed chromosomal assignment, anchoring 96.37% (1.09 Gb) of genome onto 40 pseudomolecules (0.30–198.72 Mb) (Fig. 1a, Table 1, Supplementary Material 5). The heatmap of pseudochromosome crosstalk conformed to the interaction law, confirming the assembly integrity (Fig. 1b).

**Table 1:** Statistics of the chromosome assemblies using Hi-C data

Pseudochromosome	Cluster number	Sequences length/bp
chr1	20	198,716,360
chr2	7	148,915,216
chr3	3	111,595,783
chr4	1	90,932,695
chr5	2	59,703,627
chr6	1	37,194,071
chr7	1	36,397,468
chr8	3	30,005,823
chr9	1	25,057,974
chr10	1	21,756,992
chr11	1	20,405,750
chr12	3	22,573,508
chr13	1	19,314,296
chr14	3	17,321,976
chr15	2	13,882,070
chr16	52	5,993,817
chr17	1	11,206,585
chr18	1	12,114,073
chr19	4	11,236,887
chr20	1	15,714,473
chr21	2	7,199,521
chr22	5	5,997,418
chr23	2	6,877,641
chr24	2	7,214,281
chr25	12	3,505,874
chr26	1	6,416,353
chr27	10	9,098,002
chr28	10	10,378,515
chr29	4	12,018,215
chr30	3	2,578,537
chr31	18	6,524,495
chr32	8	1,847,837
chr33	2	950,386
chr34	3	655,361
chr35	2	418,476
chr36	2	413,912
chr37	1	322,733
chr38	1	304,350
chrZ	19	83,786,400
chrW	8	13,504,621

To validate assembly accuracy, Illumina reads exhibited 99.42% alignment rate with 99.82% genome coverage using the BWA (Supplementary Material 6). Subsequently, an estimated QV score [1] was calculated using Merqury software, the result of which was 43.1876. Finally, The BUSCO analysis showed that 96.7% (single-copied gene: 96.2%, duplicated gene: 0.5%) of 8338 single-copy genes were identified as complete, 0.7% of genes were fragmented, and 2.6% of genes were missing in the assembled genome (Supplementary Material 7, Supplementary Material 2). These metrics collectively confirmed chromosomal-level integrity of the Xuefeng Black-bone chicken assembly.

### Genome annotation

423.94 Mb (37.48% genomic proportion) of repeat sequences were identified. Among these repeated sequences, 19.00% (214.91 Mb) were tandem repeats and 12.69% (143.62 Mb) were TEs (Transposable elements). These TEs were further categorized into DNA transposons (2.29%, 25.93 Mb) and retrotransposons (10.40%, 117.69 Mb). Among the retrotransposons, LINEs (long interspersed nuclear elements) were the most abundant at 7.54% of the genome, followed by LTRs (long terminal repeat elements, 2.83%) and SINEs (short interspersed nuclear elements, 0.03%) (Table 2).

**Table 2:** Summary statistics of repeat annotation in Xuefeng Black-bone chicken genome

Type	Number	Length/bp	Percentage/%
SINE	2611	373,597	0.03
LINE	283,894	85,314,411	7.54
LTR	90,619	32,002,494	2.83
DNA	120,170	25,932,616	2.29
Other	213,405	58,156,223	5.15
Unknown	35,487	7,249,269	0.64
Tandem repeats	1,053,520	214,910,367	19.00
Total		423,938,977	37.48

**Fig. 2:**
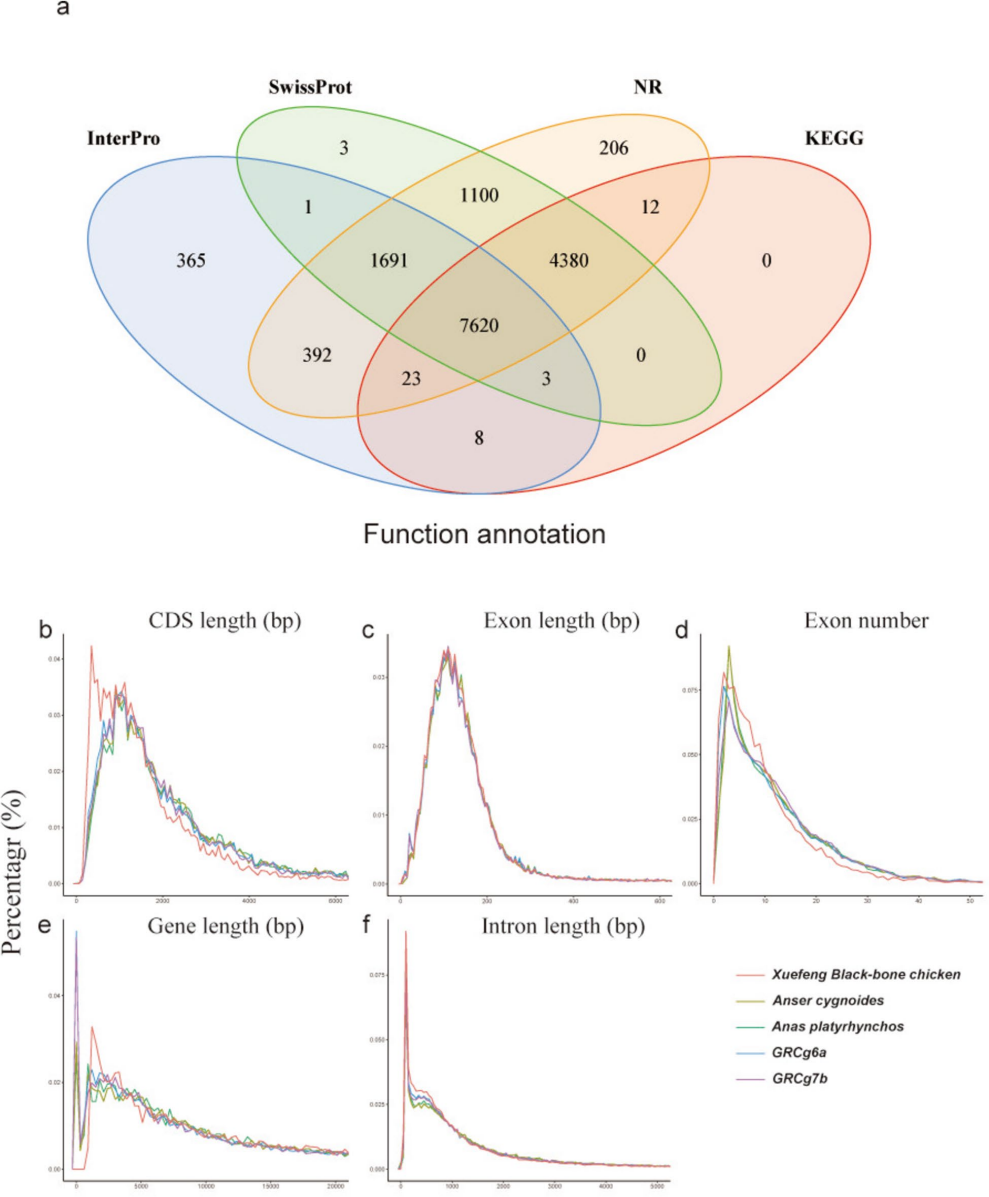
Genome annotation. **a** Venn diagram of number of genes with homology or functional classification by each method. **b**-**f** The composition of gene elements in the Xuefeng Black-bone chicken to other species. **b** CDS length. **c** Exon length. **d** Exon number. **e** Gene length. **f** Intron length

Twenty thousand one hundred forty-six protein-coding genes were identified through integrating the results of three different methods. Among these genes, 15,804 (78.45% detection rate) were annotated from the functional databases (Table 3, Fig. 2a ). The distributions of exon length, Exon length, exon number and intron length in the Xuefeng Black-bone chicken genome were found to be similar to those of closely related species (Fig. 2b-f).

**Table 3:** Functional annotation of predicted protein-coding genes

Databases	Number	Percentage/%
Nr	16,706	82.90
Swissport	15,702	77.90
KEGG	14,803	73.50
InterPro	17,523	87.00
Pfam	14,490	71.90
GO	11,640	57.80
Annotated	17,892	88.80
Unannotated	2,254	11.20
Total	20,146	100.00

For ncRNAs, a total of 291 miRNAs, 574 rRNAs, 312 snRNAs, and 294 tRNA genes were annotated (Supplementary Material 8).

### Comparative genome analysis

#### Synteny analysis

**Fig. 3:**
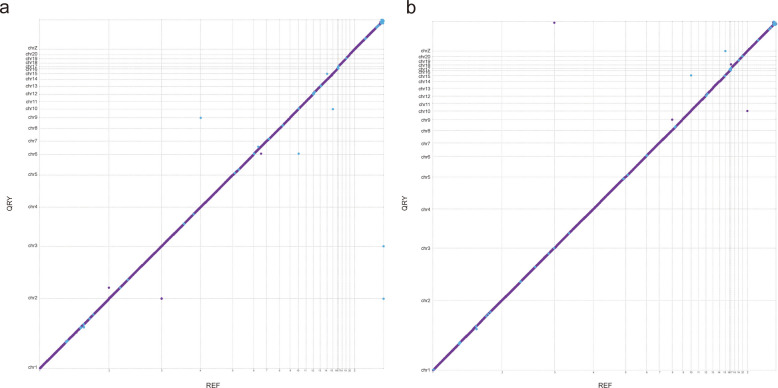
Synteny analysis. Dot-plots showing collinearity between assembled genome of Xuefeng Black-bone chicken and **a** GRCg6a and **b** GRCg7b. The x-axis denotes the chromosomes of Xuefeng Black-bone chicken. The y-axis represents genomic scaffolds of GRCg6a or GRCg7b. Diagonal line represents a good collinearity. Purple dots denote high similarity between chromosomes. Blue dots denote low similarity between chromosomes

Genome synteny analysis revealed that the majority of chromosomes in the Xuefeng Black-bone chicken genome display genomic synteny with those of GRCg6a and GRCg7b, suggesting no large-scale chromosomal rearrangements between these varieties (Fig. 3). The good genomic collinearity also attested to the high contiguity and completeness of our Xuefeng Black-bone chicken genome assembly.

#### Identification of SNPs

**Fig. 4:**
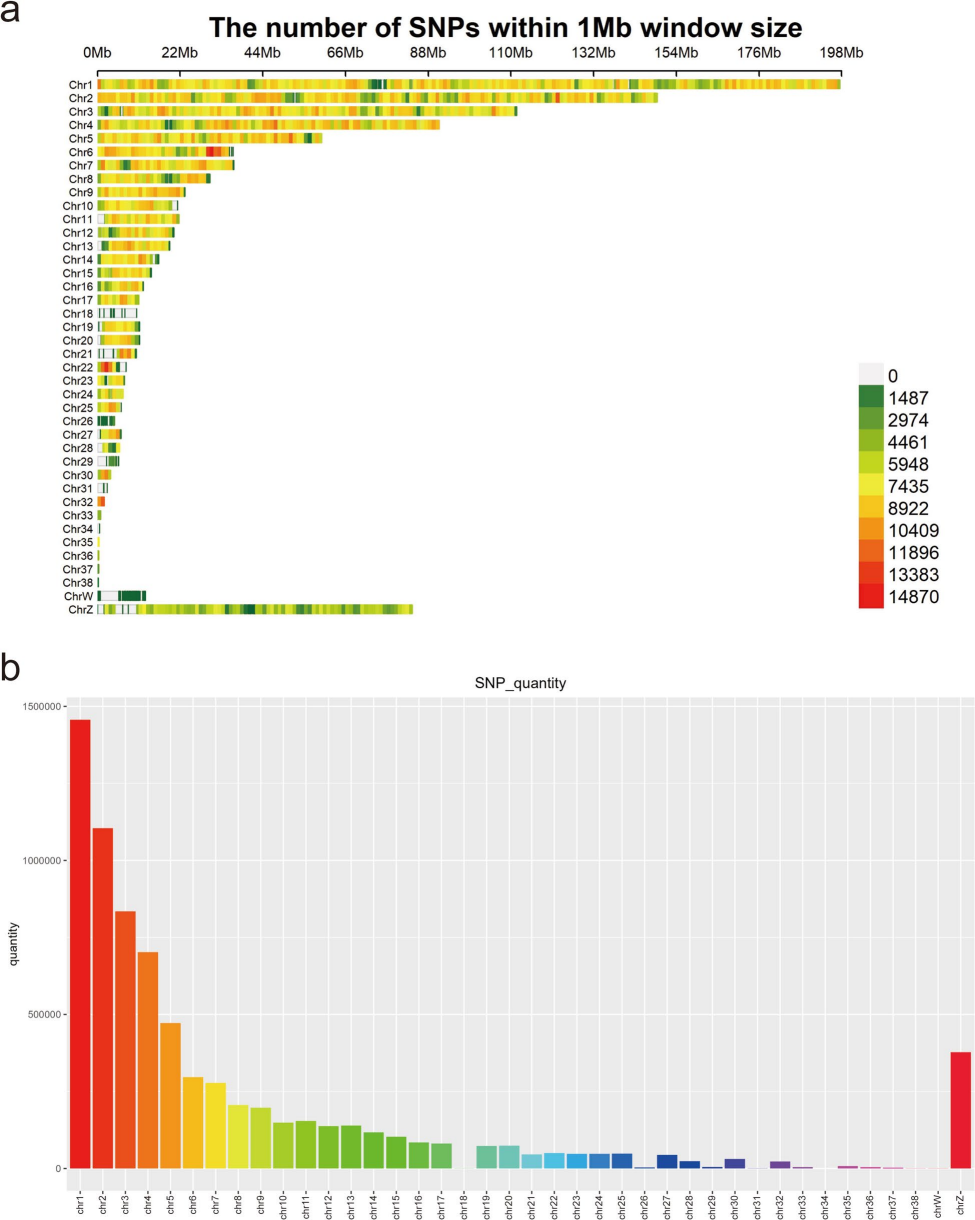
SNPs distribution on chromosomes of Xuefeng Black-bone chicken. **a** The quantity of SNPs on each chromosome. **b** Xuefeng Black-bone chicken physical map developed using GBS-SNPs. Y-axis represents each chromosome, and X-axis represents the physical positions of the SNPs on each chromosome

To study the sequence differences between Xuefeng Black-bone chicken and *Gallus gallus*, we conducted a comparative analysis of the genome sequence of Xuefeng Black-bone chicken with the reference genome sequence GRCg6a. The results showed that there was a total of 5,170,902 bases difference between the two genomes, indicating a rich presence of SNP variations. Through using a 1 Mb window to analyze the distribution of SNPs on the chromosomes, we found that SNPs were evenly distributed throughout the whole genome (Fig. 4).

#### Identification of structural variations

**Fig. 5:**
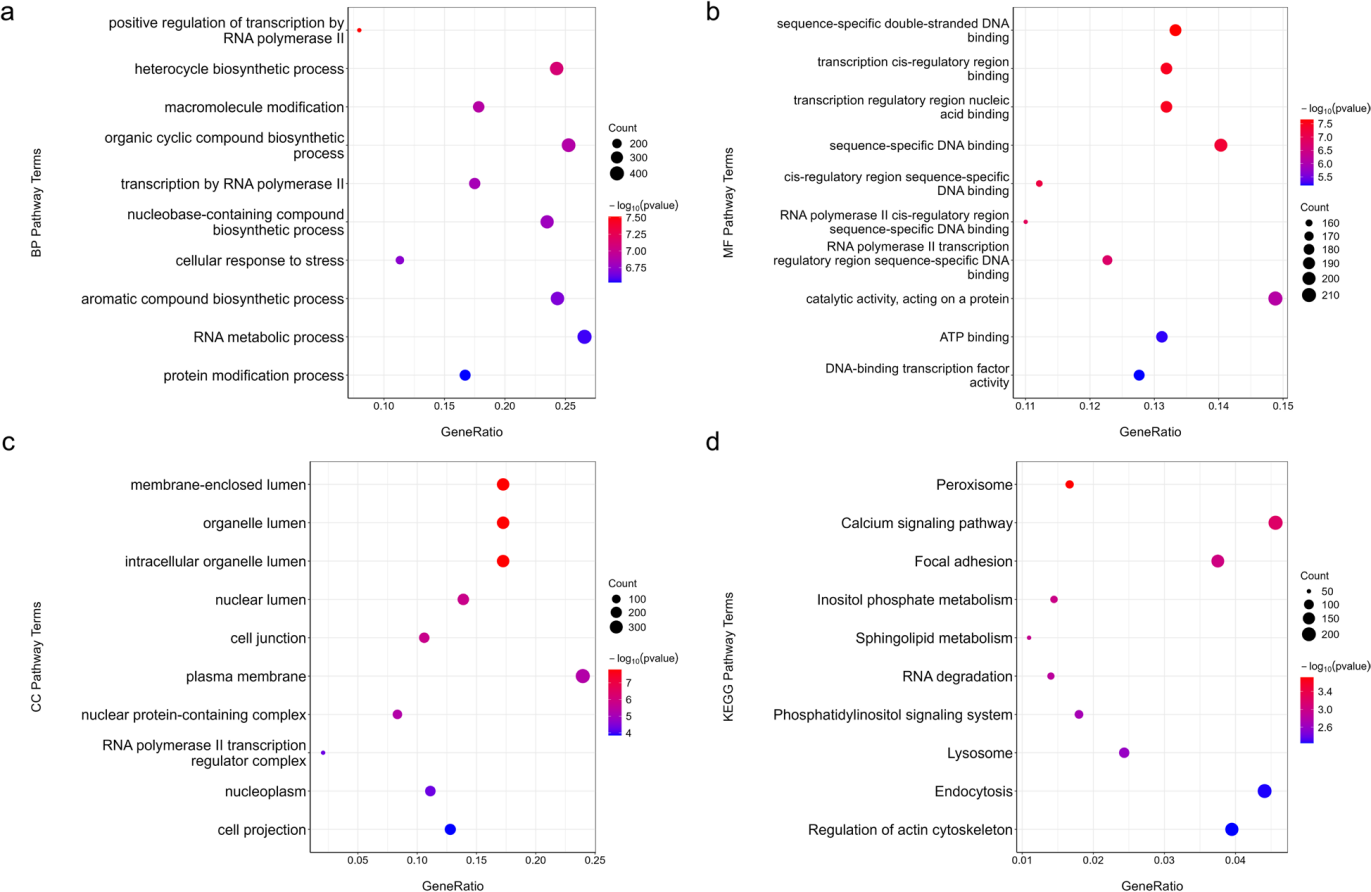
Gene functional enrichment analysis. **a**-**c** GO Enrichment Analysis. **a** Biological Process. **b** Cellular Component. **c** Molecular Function. Enrichment increased significantly from blue to purple. The larger the circle, the more significant the percentage of module genes that GO functions into the gene. **d** KEGG enrichment analysis

We identified 1127 SVs in total, including 676 insertions, 449 deletions, 0 duplications and 2 translocations. We annotated all SVs based on their genomic positions using ANNOVAR. The analysis revealed that the majority of SVs were located in intergenic regions. The distribution of SVs across specific gene-associated regions was as following: exonic regions: 11 SVs, intronic regions: 412 SVs, 5'-UTR regions: 3 SVs, 3'-UTR regions: 5 SVs, upstream flanking regions: 37 SVs, downstream flanking regions: 25 SVs. The 412 SVs located in the gene-exonic regions were linked to 11,391 genes. The complete location details for all SVs are available in the Supplementary Material 11. In order to further determine the function of these genes, GO enrichment and KEGG enrichment were performed (Fig. 5). In the biological process, the GO analysis of SV-related genes involved in positive regulation of transcription by RNA polymerase Ⅱ, heterocycle biosynthetic process and macromolecule modification (Fig. 5a); in the cell component, the genes involved in membrane-enclosed lumen, organelle lumen and intracellular organelle lumen (Fig. 5b); in the molecular function, the genes were enriched in sequence-specific double-stranded DNA binding, transcription cis-regulatory region binding and transcription regulatory region nucleic acid binding (Fig. 5c). Through the KEGG enrichment analysis, we found that the genes were mainly enriched in peroxisome, calcium signaling pathway and focal adhesion (Fig. 5d).

## Discussion

Chicken (*Gallus gallus*) serves dual roles as a primary protein source and a benchmark model in avian genomics, driving innovations across agricultural and biomedical research sectors [34]. Since the initial draft assembly of the chicken genome was released in 2004, significant advancements have been achieved in chicken genomics. These advancements have facilitated the application of various omics analyses and technologies in poultry breeding and fundamental biological research [4]. Using GRCg6a as the reference genome, we also conducted omics analyses in the study of Xuefeng Black-bone chicken. Nevertheless, the study showed that choosing reference genome for alignment can strongly implicate the downstream bioinformatics analysis, and if the chose reference genome was not the genome of the variety itself, there may be bias during alignment [11, 43, 44]. Therefore, we assembled the first chromosome-level genome of Xuefeng Black-bone chicken with the size of 1.13 Gb (contig N50 = 21.76 Mb; scaffold N50 = 83.79 Mb). The contig N50 value of the Xuefeng Black-bone chicken assemblies is higher than those of the GRCg6a, GRCg7b and GRCg7w assemblies (17.7–18.8 Mb, Supplementary Table S7) benefiting from the long reads sequencing and optimized algorithm, which demonstrated that the assembled genome is high-quality. Furthermore, we used the such criteria as degree of heterozygosity (0.65%) and repeat (18.48%), k-mer QV (43.1876), BUSCO completeness (96.7%), and chromosomal assignment rate (96.37%) to evaluate + the quality of our assembled genome, which also suggested that our assembly is the high-quality chicken genome assembly according to the criterion from the Vertebrate Genome Project [5].

Chromosome-level genomic assemblies provide indispensable frameworks for advanced genotypic-phenotypic mapping and complex trait deconvolution in avian genomic studies. Comparative genomic analysis provided the opportunity to understand characteristic phenotypes and explore the evolution of the domesticated chickens. Through comparative genomic analysis for sequence synteny and alignment, we revealed 5,170,902 SNPs and 1127 SVs between the Xuefeng Black-bone chickens and other nine chicken varieties genomes. The results of GO enrichment analysis showed that the SVs-related genes primarily involved in transcription by RNA polymerase Ⅱ, heterocycle biosynthetic process, DNA binding and KEGG enrichment analysis showed the genes were related to peroxisome, calcium signaling pathway and focal adhesion. Previous researches demonstrated that these processes were mainly associated with environmental adaption and stress resistance. For example, transcription by RNA polymerase II is recognized as a key regulatory step in eukaryotic gene expression, which coordinates cell differentiation, the maintenance of cell identity and the responses of cells to environmental changes [45, 46]. Furthermore, the genes enriched in heterocycle biosynthetic process could also confirmed that Xuefeng Black-bone chicken has the characteristic of strong stress resistance. Studies showed that the heterocyclic compounds have biological activities such as anti-inflammatory, antioxidant, anti-tumor [47]. These results suggested that the present of SVs may be associated with the strong environmental adaptability of Xuefeng Black-bone chickens.

## Conclusions

Based on Illumina short reads, PacBio SMRT long reads, and Hi-C data, we generated a chromosome-level with a size of 1.13 Gb for Xuefeng Black-bone chicken. This genome contained 40 chromosomes with contig N50 of 21.76 Mb and scaffold N50 of 83.79 Mb. Comparative genomic analyses demonstrated that there were 1127 SVs between Black-bone chicken and other chicken genomes. These SV-related genes may be associated with environmental adaption and stress resistance. The Xuefeng Black-bone chicken genome will not only promote the study of characteristic traits and evolution of Xuefeng Black-bone chicken but also guide the breeding work better.

## Supplementary Information

Supplementary Material 1. 17-mer distribution of Xuefeng Black-bone chicken genome.

Supplementary Material 2. BUSCO scores results.

Supplementary Material 3. Sequencing data used for the Xuefeng Black-bone chicken genome assembly.

Supplementary Material 4. Statistics of genome size estimation by 17-mer analysis.

Supplementary Material 5. Genomic anchor data statistics of Xuefeng Black-bone chicken.

Supplementary Material 6. Mapping rate of reads to Xuefeng Black-bone chicken genome assembly using BWA

Supplementary Material 7. BUSCO assessment of Xuefeng Black-bone chicken genome.

Supplementary Material 8. The annotated non-coding RNA in Xuefeng Black-bone chicken genome.

Supplementary Material 9. Summary of assembly indexes in several chicken genomes.

Supplementary Material 10. The related software and parameters.

Supplementary Material 11. The location of SVs

## Data Availability

The assembled genome, transcriptome, third-generation sequencing data, and second-generation sequencing data associated with this study have been deposited in the NCBI database and BioProject PRJNA1188988. These datasets are available under the following accession numbers: assembled genome JBJYYA000000000, transcriptome data SRR31629609 (Lung), SRR31629610 (Liver), SRR31629613 (Kidney), SRR31629614 (Heart), SRR31629611 (Brain), SRR31629612 (Spleen), SRR31629615 (Testis), Pacbio SMRT sequencing data SRR31628809, and Illumina sequencing data of liver SRR31629091.

## Abbreviations

CDS: Coding sequence
QV: Consensus quality value
EVM: EvidenceModeler
GO: Gene Ontology
KEGG: Kyoto Encyclopedia of Genes and Genomes
LINE: Long interspersed nuclear element
LTR: Long terminal repeat element
ncRNA: Non-coding RNA
PASA: Program to Assemble Spliced Alignment
SINE: Short interspersed nuclear element
SNP: Single nucleotide polymorphism
SV: Structural variation
TE: Transposable element
