# Supplementary figures and images for: Chromosome-level genome assembly of Xuefeng Black-bone chicken and comparative genomics analysis
Source: BMC Genomics. 2026 May 20;27:640. doi: 10.1186/s12864-026-12952-z (PMC13419013; doi:10.1186/s12864-026-12952-z)

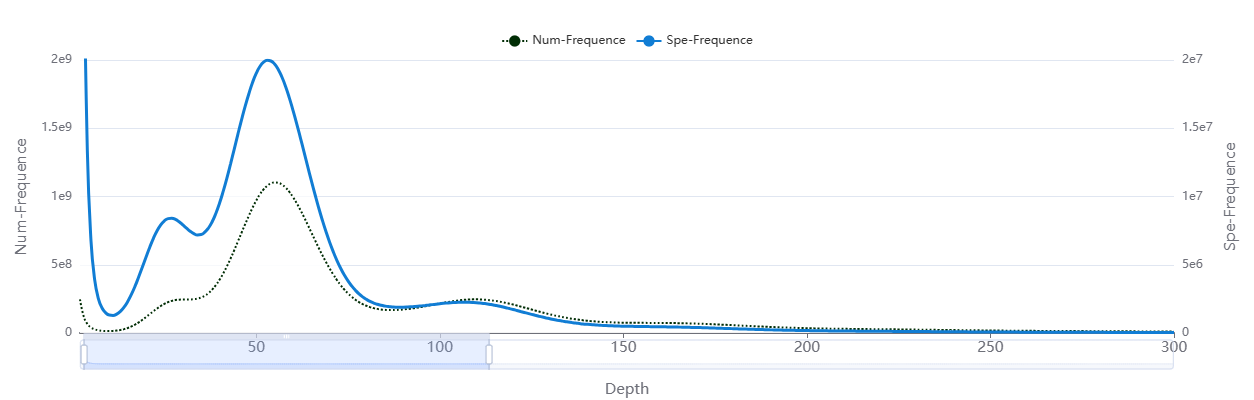

Supplement: Supplementary file 1 — Supplementary Material 1. 17-mer distribution of Xuefeng Black-bone chicken genome. [file 12864_2026_12952_MOESM1_ESM.png]

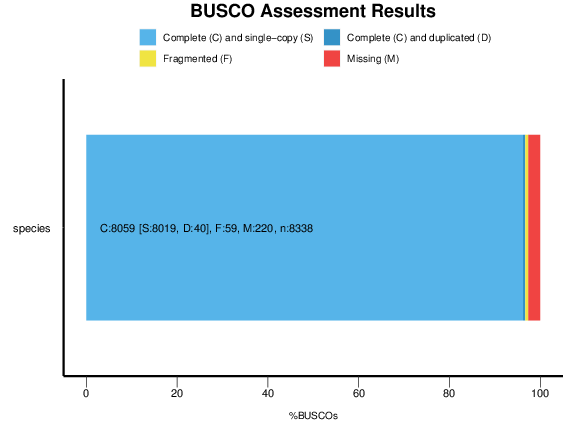

Supplement: Supplementary file 2 — Supplementary Material 2. BUSCO scores results. [file 12864_2026_12952_MOESM2_ESM.png]
