# Supplementary material for: Chromosome-level genome assembly of Xuefeng Black-bone chicken and comparative genomics analysis
Source: BMC Genomics. 2026 May 20;27:640. doi: 10.1186/s12864-026-12952-z (PMC13419013; doi:10.1186/s12864-026-12952-z)
Supplement: Supplementary file 3 — Supplementary Material 3. Sequencing data used for the Xuefeng Black-bone chicken genome assembly. [file 12864_2026_12952_MOESM3_ESM.docx]

**Table S1. Sequencing data used for the Xuefeng Black-bone chicken genome assembly**

| **Library types** | **Insert size/bp** | **Raw data/Gb** | **Clean data/Gb** | **Average read length/bp** | **Sequence coverage/X** |
| --- | --- | --- | --- | --- | --- |
| Illumina reads | 350 | 84.68 | 66.95 | 150 | 77.97 |
| HiFi reads | 15,000-18,000 | 49.66 | 49.66 | 17,281 | 45.73 |
| Hi-C reads | 350 | 150.41 | 108.49 | 150 | 138.50 |
| RNA reads | 350 | 45.08 | 43.24 | 150 | 41.51 |
| Total | - | 329.83 | 268.34 | - | 303.71 |
