# Supplementary material for: Chromosome-level genome assembly of Xuefeng Black-bone chicken and comparative genomics analysis
Source: BMC Genomics. 2026 May 20;27:640. doi: 10.1186/s12864-026-12952-z (PMC13419013; doi:10.1186/s12864-026-12952-z)
Supplement: Supplementary file 4 — Supplementary Material 4. Statistics of genome size estimation by 17-mer analysis. [file 12864_2026_12952_MOESM4_ESM.docx]

**Table S2. Statistics of genome size estimation by 17-mer analysis**

| **K-mer** | **K-mer Number** | **K-mer Depth** | **Estimated Genome Size/Mb** | **Heterozygosity** | **Repetitive sequence** |
| --- | --- | --- | --- | --- | --- |
| 17 | 59,743,616,398 | 55 | 1067.44 | 0.65% | 35.13% |
