# Supplementary material for: Chromosome-level genome assembly of Xuefeng Black-bone chicken and comparative genomics analysis
Source: BMC Genomics. 2026 May 20;27:640. doi: 10.1186/s12864-026-12952-z (PMC13419013; doi:10.1186/s12864-026-12952-z)
Supplement: Supplementary file 5 — Supplementary Material 5. Genomic anchor data statistics of Xuefeng Black-bone chicken. [file 12864_2026_12952_MOESM5_ESM.docx]

**Table S3. Genomic anchor data statistics of Xuefeng Black-bone chicken**

| **Class** | **Scaffold number** | **Total length/bp** | **Attaching rate/%** |
| --- | --- | --- | --- |
| Place | 40 | 1,090,052,372 |  |
| Unplace | 536 | 41,105,320 |  |
| Total | 576 | 1,131,157,692 | 96.37 |
