# Supplementary material for: Chromosome-level genome assembly of Xuefeng Black-bone chicken and comparative genomics analysis
Source: BMC Genomics. 2026 May 20;27:640. doi: 10.1186/s12864-026-12952-z (PMC13419013; doi:10.1186/s12864-026-12952-z)
Supplement: Supplementary file 6 — Supplementary Material 6. Mapping rate of reads to Xuefeng Black-bone chicken genome assembly using BWA [file 12864_2026_12952_MOESM6_ESM.docx]

**Table S4. Mapping rate of reads to Xuefeng Black-bone chicken genome assembly using BWA**

| **Reads** | **Mapping rate/%** | 99.42 |
| --- | --- | --- |
| **Genome** | Average sequencing depth/X | 58.35 |
|  | Coverage/% | 99.82 |
|  | Coverage at least 4X/% | 99.62 |
|  | Coverage at least 10X/% | 99.28 |
|  | Coverage at least 20X/% | 97.51 |
