# Supplementary material for: Chromosome-level genome assembly of Xuefeng Black-bone chicken and comparative genomics analysis
Source: BMC Genomics. 2026 May 20;27:640. doi: 10.1186/s12864-026-12952-z (PMC13419013; doi:10.1186/s12864-026-12952-z)
Supplement: Supplementary file 7 — Supplementary Material 7. BUSCO assessment of Xuefeng Black-bone chicken genome. [file 12864_2026_12952_MOESM7_ESM.docx]

**Table S5. BUSCO assessment of Xuefeng Black-bone chicken genome**

| **Type** | **Number** | **Percentage/%** |
| --- | --- | --- |
| Complete BUSCOs (C) | 8059 | 96.7% |
| Complete and single-copy BUSCOs (S) | 8019 | 96.2% |
| Complete and duplicated BUSCOs (D) | 40 | 0.5% |
| Fragmented BUSCOs (F) | 59 | 0.7% |
| Missing BUSCOs (M) | 220 | 2.6% |
| Total BUSCO groups searched | 8338 | - |
