# Supplementary material for: Chromosome-level genome assembly of Xuefeng Black-bone chicken and comparative genomics analysis
Source: BMC Genomics. 2026 May 20;27:640. doi: 10.1186/s12864-026-12952-z (PMC13419013; doi:10.1186/s12864-026-12952-z)
Supplement: Supplementary file 8 — Supplementary Material 8. The annotated non-coding RNA in Xuefeng Black-bone chicken genome. [file 12864_2026_12952_MOESM8_ESM.docx]

**Table S6. The annotated non-coding RNA in Xuefeng Black-bone chicken genome**

| **Type** | | **Copy (number)** | **Average-length/bp** | **Total-length/bp** | **Percent of the genome/%** |
| --- | --- | --- | --- | --- | --- |
| **miRNA** | | 291 | 86.660 | 25,218 | 0.002 |
| **tRNA** | | 294 | 75.354 | 22,154 | 0.002 |
| **rRNA** | rRNA | 574 | 446.106 | 256,065 | 0.023 |
|  | 18S | 104 | 836.798 | 87,027 | 0.008 |
|  | 28S | 376 | 416.178 | 156,483 | 0.014 |
|  | 5.8S | 47 | 153.255 | 7203 | 0.001 |
|  | 5S | 47 | 113.872 | 5352 | 0.000 |
| **snRNA** | snRNA | 312 | 122.926 | 38,353 | 0.003 |
|  | CD-box | 123 | 90.813 | 11,170 | 0.001 |
|  | HACA-box | 78 | 140.474 | 10,957 | 0.001 |
|  | splicing | 93 | 140.903 | 13,104 | 0.001 |
|  | scaRNA | 15 | 187.400 | 2811 | 0.000 |
|  | Unknown | 3 | 103.667 | 311 | 0.000 |
