# Supplementary material for: Chromosome-level genome assembly of Xuefeng Black-bone chicken and comparative genomics analysis
Source: BMC Genomics. 2026 May 20;27:640. doi: 10.1186/s12864-026-12952-z (PMC13419013; doi:10.1186/s12864-026-12952-z)
Supplement: Supplementary file 9 — Supplementary Material 9. Summary of assembly indexes in several chicken genomes. [file 12864_2026_12952_MOESM9_ESM.docx]

**Table S7. Summary of assembly indexes in several chicken genomes**

| **Varieties** | **Assembly strategies** | **Assembly Size/Gb** | **Scaffold N50/Mb** | **Contig N50/Mb** | **BUSCO/%** | **Number of chromosomes** |
| --- | --- | --- | --- | --- | --- | --- |
| Xuefeng Black-bone chicken | PacBio + Illumina + Hi-C | 1.13 | 83.79 | 21.76 | 96.7 | 40 |
| GRCg7b | PacBio + Illumina + Hi-C + Bionano | 1.1 | 90.9 | 18.8 | 98.6 | 41 |
| GRCg7w | PacBio + Illumina + Hi-C + Bionano | 1.1 | 90.6 | 17.7 | 98.7 | 41 |
| GRCg6a | Pacific Biosciences RSII | 1.1 | 20.8 | 17.7 | - | 34 |
