# Supplementary material for: Chromosome-level genome assembly of Xuefeng Black-bone chicken and comparative genomics analysis
Source: BMC Genomics. 2026 May 20;27:640. doi: 10.1186/s12864-026-12952-z (PMC13419013; doi:10.1186/s12864-026-12952-z)
Supplement: Supplementary file 10 — Supplementary Material 10. The related software and parameters. [file 12864_2026_12952_MOESM10_ESM.docx]

**Table S8. The related software and parameters**

| **Analysis module** | **Software** | **Version** | **Parameter** |
| --- | --- | --- | --- |
| Sequencing | CCS | V6.4.0 | min-passes=3, min-rq=0.99 |
|  | Hi-C-Pro | V3.1.0 | default |
|  | Fastp | V0.23.1 | default |
| Survey analysis | Jellyfish | V2.2.7 | default |
|  | SOAPdenovo2 | r242 | default |
| Genome assembly | Hifiasm | V0.13.0-R307 | default |
|  | ALLHiC | V0.9.8 | --minREs 50 --maxLinkDensity 3 --nonInformativeRatio 2 |
|  | Juicebox | V1.11.08 |  |
| Genome quality assessment | BUSCO | V.5.7.1 | default |
|  | BWA | V0.7.18 | default |
|  | Merqury | V1.3 | default |
| Repetitive sequence annotation | RepeatModeler | V2.0.3 | -engine ncbi -pa 30 -LTRStruct |
|  | RepeatMasker | V4.1.2-P1 | -nolow -no_is -norna -pa 30 |
|  | blast | V2.2.26 | -e 1e-10 -v 10000 -b 10000 |
|  | Tandem Repeat Finder | V4.10.0 | 2 7 7 80 10 50 500 |
| Non-coding RNA annotation | cmsearch | V1.1.4 | default |
|  | tRNAscan-SE | V1.4 | default |
|  | Rfam | V14.1 | default |
|  | TBLASTN | v2.2.26 | E-value ≤ 1e−5 |
|  | Trinity | V2.8.5 | --normalize_reads  --full_cleanup  --min_glue 2  --min_kmer_cov 2 |
| Protein-coding genes annotation | Hisat2 | V2.2.1 | default |
|  | Stringtie | V2.2.1 | default |
|  | Augustus | V3.5 | --species=pasa1  --uniqueGeneId=TRUE  --noInFrameStop=TRUE  --GFF3=on  --genemodel=complete  --strand=both |
|  | Snap | V2013.11.29 | -gff pasa1.hmm |
|  | Blastall | V2.2.26 | -e 1e-05 -F T -m 8 |
|  | Solar | V0.9.6 | -a prot2genome2 -z -f m8 |
|  | Genewise | V2.4.1 | -tfor -genesf -gff -sum |
|  | EVM | V1.1.1 | --segmentSize 200000  --overlapSize 20000  --min_intron_length 20 |
|  | Interproscan | V5.59-91.0 | -cpu 20 -format tsv -appl  ProDom,SMART,ProSiteProfiles,  PRINTS,Pfam,Panther -iprlookup  -dp -goterms |
|  | Blastp | V2.2.26 | -max_target_seqs 1 -evalue 1e-4 |
| Functional annotation | Diamond | V0.8.22 | --more-sensitive -k 10 -e 1e-5 -f 6  qseqidqlen qstart qend sseqid  slen sstart sendpident ppos  qcovhsp bitscore evalue  --salltitles --threads 10 |
|  | MUMmer4 | V4.00 | –c 1000  -1 -i90 -l10000 |
|  | MCScanX |  | default |
| Synteny analysis | Winnowmap2 | V2.03 | -a -x asm20 --cs -r 2000 -k 15 |
|  | Svim-asm | V1.0.3 | default |
| Structural Variations Analysis | SURVIVOR | V1.0.7 | 1000 7 1 1 0 50 |
|  | KOBAS | V3.0 | default |
|  |  |  |  |
|  |  |  |  |
